# Acute and Chronic Cardiovascular Manifestations of COVID-19: Role for Endotheliopathy

**DOI:** 10.14797/mdcvj.1044

**Published:** 2021-12-15

**Authors:** John P. Cooke, John H. Connor, Abhishek Jain

**Affiliations:** 1 Houston Methodist Research Institute, Houston Methodist, Houston, TX, US; 2 Boston University Medical Center and National Emerging Infectious Diseases Laboratories, Boston University, Boston, MA, US; 3 Texas A&M University, College Station, TX, US; 4 Texas A&M Health Science Center, Bryan, TX, US

**Keywords:** SARS-CoV-2, endothelium, deep venous thrombosis, pulmonary embolism, myocardial infarction, cerebrovascular attack, dementia, myocarditis, nitric oxide

## Abstract

SARS-CoV-2, the virus that causes coronavirus disease 19 (COVID-19), is associated with a bewildering array of cardiovascular manifestations, including myocardial infarction and stroke, myocarditis and heart failure, atrial and ventricular arrhythmias, venous thromboembolism, and microvascular disease. Accumulating evidence indicates that a profound disturbance of endothelial homeostasis contributes to these conditions. Furthermore, the pulmonary infiltration and edema, and later pulmonary fibrosis, in patients with COVID-19 is promoted by endothelial alterations including the expression of endothelial adhesion molecules and chemokines, increased intercellular permeability, and endothelial-to-mesenchyme transitions. The cognitive disturbance occurring in this disease may also be due in part to an impairment of the blood-brain barrier. Venous thrombosis and pulmonary thromboembolism are most likely associated with an endothelial defect caused by circulating inflammatory cytokines and/or direct endothelial invasion by the virus. Endothelial-targeted therapies such as statins, nitric oxide donors, and antioxidants may be useful therapeutic adjuncts in COVID-19 by restoring endothelial homeostasis.

## Introduction

The endothelium is a diaphanous film of tissue that invests the luminal surface of all blood vessels with a nonthrombogenic lining. The endothelium is critically important in vascular patency and homeostasis. It generates factors that prevent platelet adherence and aggregation, suppresses leukocyte adhesion and infiltration, relaxes the vascular smooth muscle to permit the smooth flow of blood, and inhibits abnormal growth of vascular cells to prevent intimal and medial hyperplasia. The healthy endothelium also governs the exchange of gases, nutrients, fluids, and metabolites into organs and tissues. Thus, a healthy endothelium is critical for normal cardiovascular structure and function. Viral impairment of endothelial function has been noted in multiple acute viral diseases, including coronavirus disease 19 (COVID-19, caused by the SARS-CoV-2 virus). This endothelial disruption could cause or contribute to a number of cardiovascular disorders involving thrombosis, inflammation, altered vascular permeability, contractility, and proliferation.

## A Role for Endotheliopathy in Acute Cardiovascular Events

Accumulating evidence indicates that the cardiovascular manifestations of COVID-19 are likely due to a profound endothelial alteration secondary to circulating inflammatory cytokines and/or direct viral invasion of the endothelium^1,2,3,4^ as well as infection of supporting cells (eg, pericytes).^5,6^ Viral inclusion bodies have been observed by electron microscopy in tissues from patients succumbing to SARS-CoV-2, and circulating inflammatory factors in infected patients cause systemic impairment of endothelial function.^7,8^ A viral induction of endothelial pathology would be expected to underlie many of the cardiovascular manifestations of COVID-19. Indeed, endothelial dysfunction is a known independent risk factor for heart attack and stroke. All known risk factors for cardiovascular disease (eg, hypertension, diabetes mellitus, hypercholesterolemia) cause endothelial dysfunction long before there is any histopathological evidence of vascular disease.^9,10,11^ Genetic induction of endothelial dysfunction (as with endothelial nitric oxide synthase knockout mice) accelerates vascular disease.^12,13^

The hypothesis that an endotheliopathy underlies COVID-19 is supported by prior observations that other viral illnesses can induce endothelial dysfunction, and these viral illnesses are associated with later cardiovascular disease.^14,15,16,17,18,19,20,21^ For example, prior infection with influenza is known to be associated with cardiovascular disease. In this regard, it is notable that post-mortem tissues from patients succumbing to influenza or COVID-19 show greater endothelial damage from SARS-CoV-2 infection than with H1N1 infection.^22^ Of concern, we have shown that viral activation of cell-autonomous innate immune activation induces epigenetic alterations that facilitate nuclear reprogramming to a different cell phenotype or lineage,^23,24,25,26,27^ with a loss of normal cellular functions that maintain homeostasis. As discussed below, such epigenetic changes may have long-term effects and contribute to post-COVID syndrome and later cardiovascular disease.

**Figure 1:**
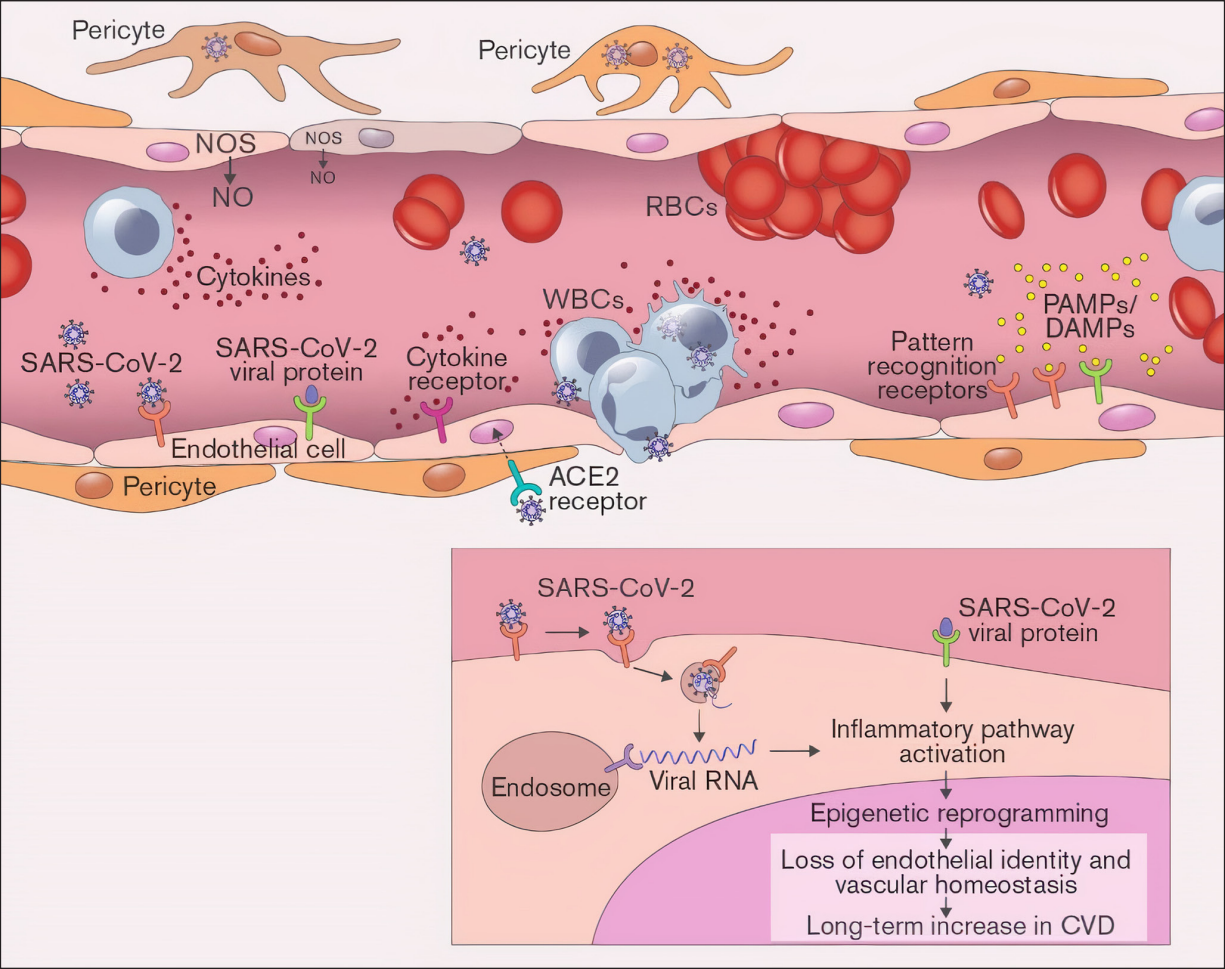
SARS-CoV-2 activates inflammatory signaling within endothelial cells (EC) that alters endothelial homeostasis and promotes inflammation, vascular permeability, and thrombosis. Epigenetic alterations contribute to the acute and chronic manifestations of COVID endotheliopathy. NOS: nitric oxide synthase; NO: nitric oxide; WBCs: white blood cells; RBCs: red blood cells; PAMP: pathogen-associated molecular patterns; DAMP: damage-associated molecular patterns; CVD: cardiovascular disease.

The endotheliopathy induced by COVID-19 contributes to a hyperinflammatory response and hypercoagulability (***Figure 1***). Pattern recognition receptors (PRRs) on the endothelium can sense pathogen-associated molecular patterns presented by the virus as well as damage-associated molecular patterns generated by damaged cells. Activation of these endothelial PRRs stimulates an inflammatory response that causes loss of the normal vasodilatory factors such as nitric oxide (NO) and prostacyclin. Loss of these factors also facilitates platelet and leukocyte adhesion. Interaction of blood elements with the vessel wall is aggravated by the expression of endothelial adhesion molecules, chemokines, and inflammatory cytokines that precipitate a local inflammatory response. This acute endotheliopathy and cardiovascular injury is expected to be more severe in older individuals with preexisting endothelial dysfunction and cardiovascular disease.^28,29,30^ The increasing impairment of endothelial function with age contributes to the increased vulnerability of the elderly to SARS-CoV-2.

Depending on which vascular circulation is affected, the endothelial disturbance created by viral invasion and circulating cytokines could promote pulmonary inflammation and edema, microvascular inflammation and myocarditis, coronary thrombosis and myocardial infarction, carotid artery thrombosis and stroke, peripheral microvascular occlusion manifesting as local tissue ischemia and inflammation (eg, “COVID toes”), and venous thrombosis and pulmonary embolism. In addition to precipitating acute cardiovascular events, dysfunction of the cerebrovascular endothelium could cause or contribute to cognitive impairment.^31^ Notably, cardiovascular manifestations of COVID-19 can occur in the absence of respiratory symptoms.^28^

## Venous Thrombosis as a Consequence of Covid-19 Endotheliopathy

Because the other acute cardiovascular manifestations of COVID-19 are discussed elsewhere in this issue, we highlight here the venous thrombosis that may occur in COVID-19.

Deep venous thrombosis (DVT) and its consequences (venous thromboembolism, or VTE) contributes to 100,000 deaths annually in the US.^32^ In contrast to arterial thrombosis, DVT research has received less attention, prompting the American Surgeon General to issue a Call to Action in 2008 to stimulate DVT research.^33^ Fast forward to 2020, DVT is now recognized as one of the life-threatening manifestations in patients with COVID-19 and a major determinant of mortality.^34^ A study of patients with severe COVID-19 in Wuhan, China, revealed that nearly 90% had lower-extremity DVT, and those with DVT had worse survival rates.^34^ Before the use of systemic anticoagulation in hospitalized COVID patients, the majority of those succumbing to SARS-CoV-2 had DVT, and it contributed to the demise of at least one-third of patients.^35^

Notably, COVID-19 is associated with endothelial activation and damage in several organs.^6,36^ SARS-CoV-2 invades endothelial cells in an organoid culture,^37^ and its viral inclusion bodies are seen in endothelial cells at autopsy. Autopsy findings of COVID-19 patients have further revealed changes in endothelial cytoarchitecture and apoptosis as well as microthrombi and larger clots in the lungs, kidneys, and mesenteric vessels—all manifestations of the virus’ vasculotropic characteristics.^6^ Endothelial dysfunction and venous thrombosis contribute to respiratory failure in COVID-19.^38,39^ These findings emphasize that endotheliopathy—and vascular thrombosis in particular—is a common aspect of severe COVID-19. Still understudied is the mechanism by which this occurs and how it might be best mitigated in ways that limit both short- and long-term consequences of this damage.

## Virchow’s Triad and Sars-cov-2

The venous thrombosis observed in COVID-19 is due to the interaction of the three determinants of thrombosis (Virchow’s triad): vascular injury, stasis, and blood coagulability. In this case, the vascular injury is a viral-induced endotheliopathy. The stasis in the limb veins is a consequence of the immobile hospitalized patient but also the complex hemodynamics in the vicinity of the valve cusps. The increased blood coagulability is associated with immune-mediated mechanisms generated during the response to the infection.^40^

There is an unmet need to understand the effects of the virus and/or blood-borne inflammatory cytokines on the endothelium, understand how these effects interact with the uniquely complex venous hemodynamics (mechanotransduction), and discover endothelium-stabilizing strategies that may be cooperatively therapeutic with anticoagulants. There is a dearth of tools for investigating how these thrombi develop. Cell monolayer or simple organoid growth does not reproduce the key architecture that drives thrombosis. Most of these models forego inclusion of venous valves when it is well-known that DVT is primarily initiated at the site of venous valve pockets by endothelial disruption, blood stasis, and/or hypercoagulable blood—the three factors known as Virchow’s triad.^41,42^

**Figure 2:**
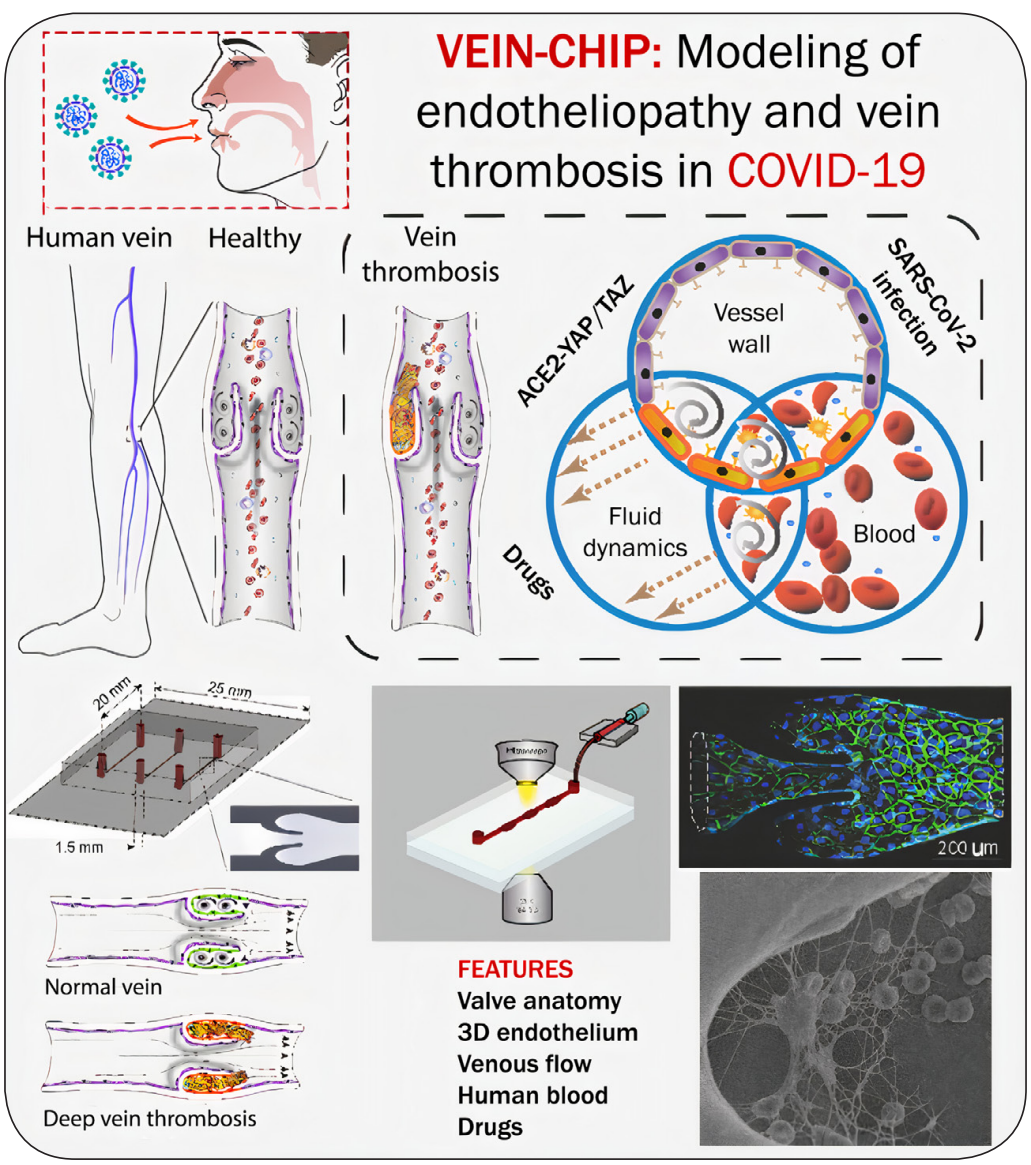
Infographic describing how the vein-chip will model endotheliopathy and thrombosis in COVID-19. Bottom fluorescence micrograph and scanning electron image taken from Rajeeva et al. Microengineered Human Vein-Chip Recreates Venous Valve Architecture and Its Contribution to Thrombosis. Small. 2020;16(49):2003401. doi: *10.1002/smll.202003401*

To understand the interactions of these elements of Virchow’s triad during COVID-19 infection, three organizations—including Texas A&M University, the Houston Methodist Research Institute, and Boston University—have teamed together to bring complementary expertise in vein-chip bioengineering and innovation (Jain), molecular and computational biology of endothelial function and cell fate (Cooke), and techniques to define host response to coronaviral infection (Connor). Springing from this multidisciplinary collaboration and an urgency to address the impact of COVID-19, our objective is to understand the determinants of SARS-CoV-2–induced venous thrombosis; determine the roles of endothelial, hemodynamic, and humoral alterations; and propose therapeutic strategies. We intend to elucidate the mechanisms of COVID-19–associated venous thrombosis using our vein-on-a-chip (***Figure 2***).^43^ This vein chip is an endothelialized device incorporating the structure and unique hemodynamics proximal and distal to the venous valve cusp. We have used this chip to determine the role of major determinants of venous thrombosis—endothelium, hemodynamics, and blood components (Virchow’s triad).^41,42^ A salient feature of our technology is that it reveals differential endothelial phenotypes that are clinically observed but not mechanistically understood by other preclinical models.

**Figure 3:**
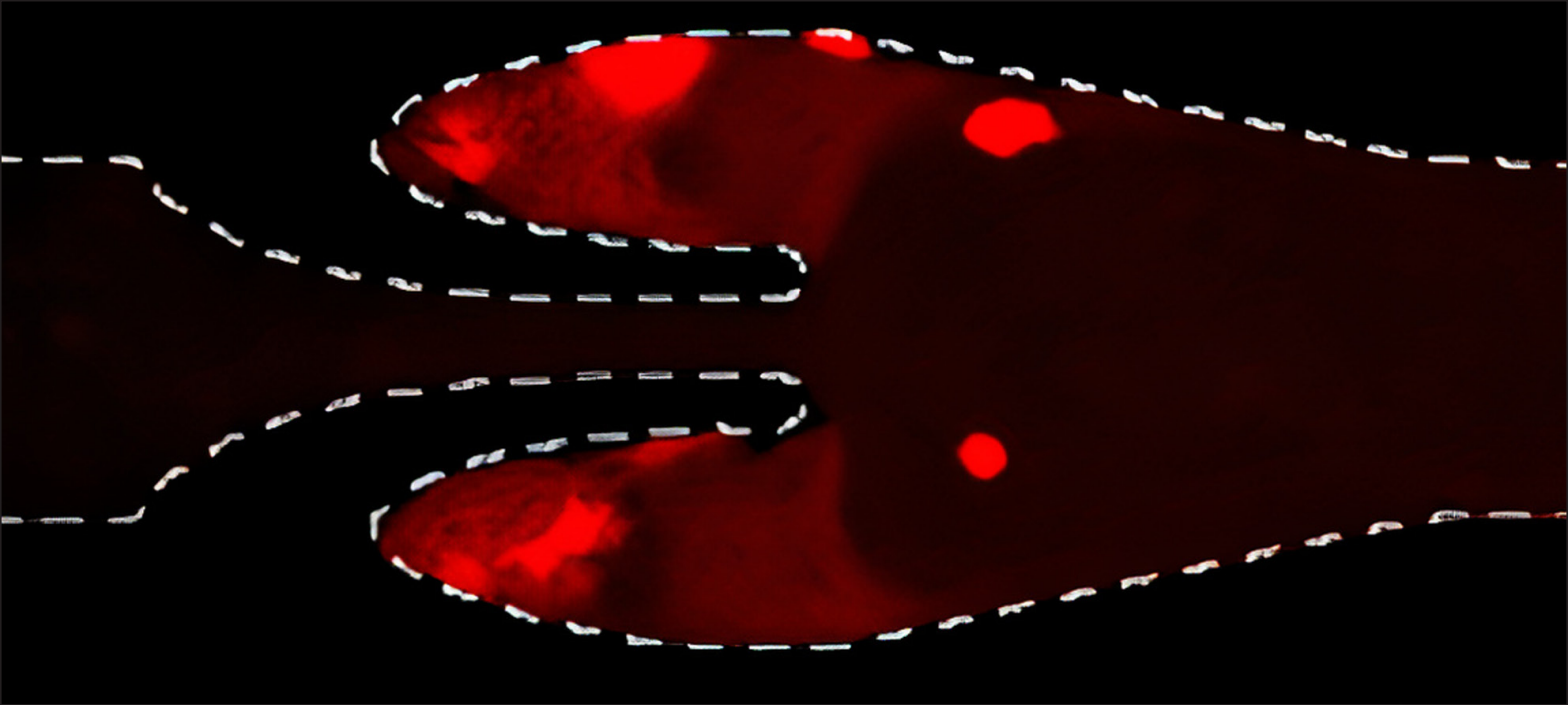
A section of vein-chip. Human vein-chip more accurately models human venous architecture, flow, and thrombosis. Taken from Rajeeva et al. Microengineered Human Vein-Chip Recreates Venous Valve Architecture and Its Contribution to Thrombosis. Small. 2020;16:2003401. doi: *10.1002/smll.202003401*. Image enlarged using Let’s Enhance.

The most common animal model of thrombosis is the mouse inferior vena cava model.^44,45^ Typically, venous thrombosis is induced chronically by inducing stasis or stenosis (with a ligature) or rapidly by an acute injury (eg, using chemical injury) of the inferior vena cava. While these models have decoded several key mechanisms that govern DVT, the lack of valve function (***Figure 3***, middle panel) and genetic differences with respect to humans can limit them in studying Virchow factors.^43,46^ Existing animal models of DVT are expensive and suboptimal in that they are not amenable to a reductionist approach.^47^ In contrast, our vein-chip microfluidic cell culture platform offers a new in vitro tool for studying how endothelial cells, humoral substances, and hemodynamics interact to influence human vein diseases or respond to therapeutics.^48,49^ Using such blood-vessel chips, we have elucidated mechanisms of thromboinflammation and identified potential therapeutic approaches in areas where animal models have shown limitations.^44,50,51,52,53,54^ With this multidisciplinary team, together with federal funding and corporate collaboration, we intend to refine therapeutic strategies for preventing and treating virally-induced venous thrombosis.

## Post Covid-19 Condition and Endothelial Epigenetics

Some individuals have persistent effects of SARS-CoV-2 infection long after they are serologically negative for the virus and have converted to immunopositivity. In early October 2021, the World Health Organization (WHO) classified these “long haulers” as having “post COVID-19 condition,” with symptoms of physical fatigue, tachycardia, and dyspnea with little exertion as well as cognitive impairment that interferes with activities of daily life.^55,56,57^ According to the WHO, the symptoms typically initiate within 3 months after probable or confirmed SARS CoV-2 infection and last at least 2 months. Although it is difficult to determine the exact number of patients who experience post COVID-19 condition, data indicate that roughly 10% to 20% continue to experience symptoms after acute infection. By comparison, recovery from influenza is complete within 2 weeks in over 90% of cases. The symptoms of those with post COVID-19 condition could be explained in part by a severe and global endothelial dysfunction that impairs pulmonary, coronary, cerebral, and skeletal muscle microvasculature.

**Figure 4:**
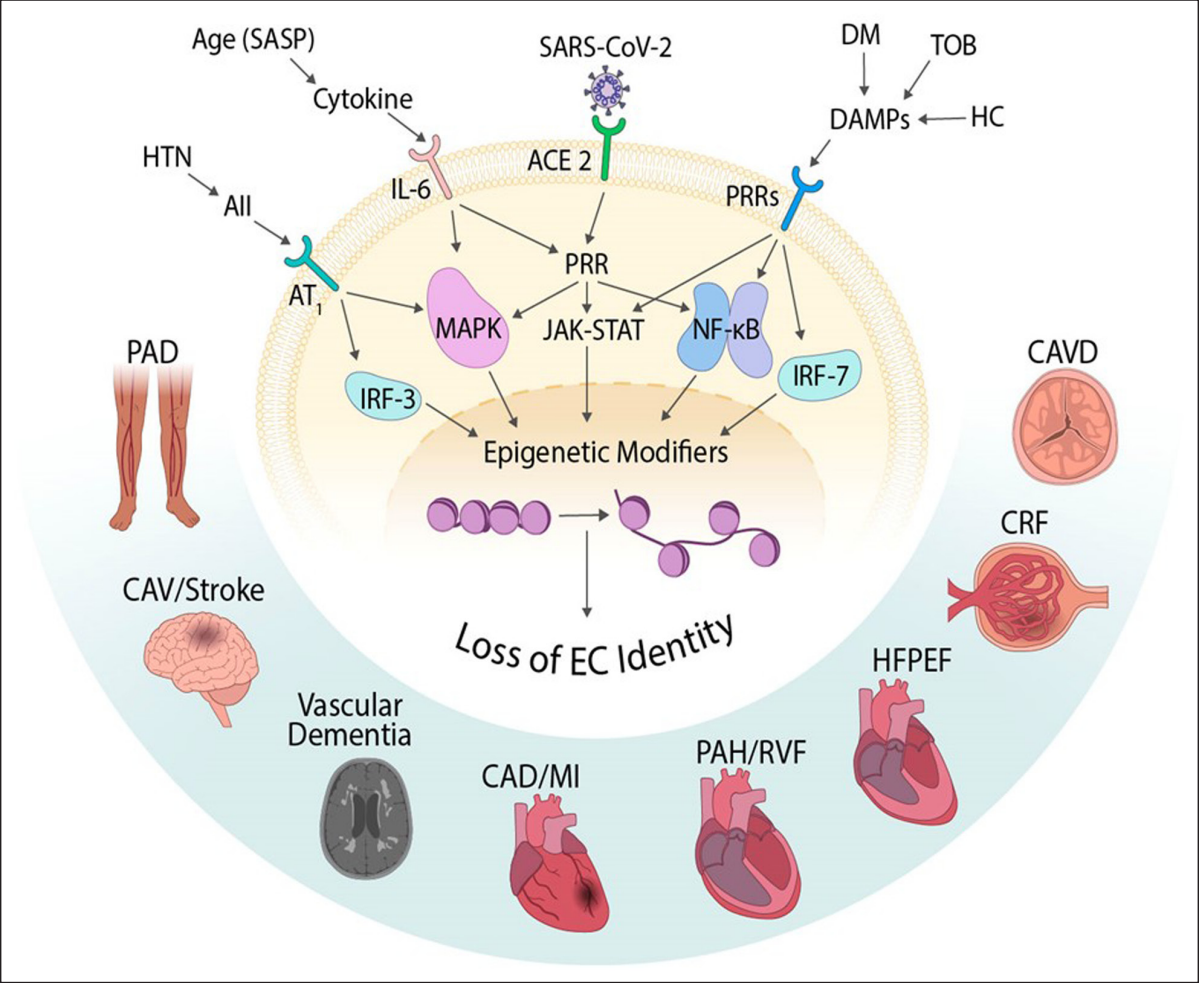
Pathways in endothelial cells (ECs) that contribute to epigenetic alterations and EC loss of function causing downstream cardiovascular disease. ACE2: angiotensin converting enzyme 2; DM: diabetes mellitus; TOB: tobacco exposure; DAMP: damage-associated molecular patterns; HC: hypercholesterolemia PRR: pattern recognition receptors; JAK/STAT: Janus kinase signal transducer and activator of transcription; NF-kB: nuclear factor kappa B; IRF-7: interferon regulatory factor 7; CAVD: calcific aortic valve disease; CRF: corticotropin-releasing factor; HFPEF: heart failure with preserved ejection fraction; PAH/RVP: pulmonary arterial hypertension/right ventricular pressure; CAD/MI: coronary artery disease/myocardial infarction; CAV: cardiac allograft vasculopathy; PAD: peripheral arterial disease; HTN: hypertension.

As described above, COVID-induced endotheliopathy likely participates in the acute manifestations of the disease since it would likely exacerbate the inflammatory state, prothrombotic state, and vascular permeability—each of which may contribute to the many disease manifestations. However, what is not known, and what has not been anticipated nor adequately discussed, is the likely effect of a persistent epigenetic alteration of the endothelium (***Figure 4***). Specifically, epigenetic alterations in genes that regulate endothelial cell identity are likely responsible for the acute impairment or loss of normal endothelial function associated with SARS-CoV-2 infection. Furthermore, the persistence of these epigenetic alterations would be expected to cause a chronic impairment in endothelial cell function that would cause or contribute to many of the symptoms of long COVID. Chronic impairment of endothelial function would be expected to accelerate atherosclerosis and increase the risk of later arterial occlusive disease in the coronary or cerebrovascular circulations.

Viral invasion of endothelial cells, or exposure to inflammatory cytokines, are triggering events that activate cell-autonomous innate immune signaling in endothelial cells, which in turn may diminish or erase epigenetic determinants of endothelial identity. In the short term, this postulated endotheliopathy would be expected to cause further inflammation, vasoconstriction, and coagulation. In the long term, a persistent epigenetic alteration will promote endothelial activation manifested by endothelial adhesion molecules and chemokines, which will aggravate vascular inflammation and progression of atherosclerosis and other vascular diseases. Furthermore, in some organs this attenuation of endothelial identity will also promote endothelial-to-mesenchyme transition, leading to rarefaction of the microvasculature and disseminated fibrosis in multiple organs. Thus, a loss of endothelial identity would be expected to (1) accelerate atherosclerosis manifested as coronary, cerebrovascular, and peripheral arterial disease; (2) cause excess morbidity and mortality from strokes, vascular dementia, myocardial infarction, and critical limb ischemia; (3) generate cardiac, pulmonary, and renal fibrosis, with impaired perfusion and function of these organs; (4) increase valvular and vascular calcification; (5) increase the prevalence of hypertension and chronic renal failure; and (6) cause a persistent abnormality of the cerebral microvasculature contributing to cognitive impairment.^31^

Notably, endotheliopathy related to a traumatic event can persist and accelerate cardiovascular disease. Longitudinal studies have shown that individuals who were born prematurely have endothelial dysfunction as children that persists into young adulthood and a greater risk of cardiovascular disease in adulthood.^58,59^ These children have greater risk of infections in infancy and childhood,^60^ and this increase in infection exposure has been tied to later cardiovascular disease.^61^ Similarly, exposure to secondhand smoke in childhood is associated with increased risk of carotid plaque in adulthood.^62^ Recent studies reveal that activation of damage- and pathogen-associated molecular patterns cause epigenetic changes that place a cell into a state of phenotypic fluidity.^23,24,25,26,27^ Of course, this phenomenon can be useful in defense against a pathogen or in response to injury. However, this same inflammatory signaling can facilitate a phenotypic switch to a deleterious phenotype, depending on the context.

The hopeful news is that, despite epigenetic alterations that may persist in the absence of countervailing forces, there are potential therapeutic approaches that may reverse the aberrant epigenetic markings and restore normal endothelial cell identity and function, thereby preventing cardiovascular disease. A comprehensive characterization of COVID-19–associated endotheliopathy, and an understanding of the mechanisms of acute and chronic endothelial alterations induced by SARS-CoV-2, will lead to an improved understanding of the many manifestations of COVID-19 and a refined management approach for this and other vasculotropic viral diseases.

## Key Points

- Endothelial homeostasis is critical for normal cardiovascular structure and function.
- Inflammatory cytokines generated during SARS-CoV-2 infection, and/or viral entry into endothelial cells, cause vascular impairment resulting in vascular inflammation, edema, and thrombosis.
- An endotheliopathy likely contributes to the pulmonary and cardiovascular manifestations of COVID-19.
- Some of the unusual manifestations of COVID-19 including myocarditis, cognitive disturbance, and “COVID toes” may be driven by endothelial perturbation.
- Post COVID-19 condition may be due to a persisting endothelial dysfunction.
- Endothelial-targeted therapies such as statins, angiotensin converting enzyme inhibitors, angiotensin receptor blockers, and nitric oxide donors may be useful in this viral disorder.

## CME Credit Opportunity

Houston Methodist is accredited by the Accreditation Council for Continuing Medical Education (ACCME) to provide continuing medical education for physicians.

Houston Methodist designates this enduring material for a maximum of .25 AMA PRA Category 1 Credit™. Physicians should claim only the credit commensurate with the extent of their participation in the activity.

Click to earn CME credit: *https://journal.houstonmethodist.org/articles/10.14797/mdcvj.1044/*

## References

[1] Teuwen LA, Geldhof V, Pasut A, Carmeliet P. COVID-19: the vasculature unleashed. Nat Rev Immunol. 2020 Jul;20(7):389–391. doi: 10.1038/s41577-020-0343-0 32439870 PMC7240244

[2] Libby P, Lüscher T. COVID-19 is, in the end, an endothelial disease. Eur Heart J. 2020 Sep 1;41(32):3038–3044. doi: 10.1093/eurheartj/ehaa623 32882706 PMC7470753

[3] Iba T, Connors JM, Levy JH. The coagulopathy, endotheliopathy, and vasculitis of COVID-19. Inflamm Res. 2020 Dec;69(12):1181–1189. doi: 10.1007/s00011-020-01401-6 32918567 PMC7486586

[4] Nagashima S, Mendes MC, Camargo Martins AP, et al. Endothelial Dysfunction and Thrombosis in Patients With COVID-19-Brief Report. Arterioscler Thromb Vasc Biol. 2020 Oct;40(10):2404–2407. doi: 10.1161/ATVBAHA.120.314860 32762443 PMC7505138

[5] He L, Mäe MA, Muhl L, et al. Pericyte-specific vascular expression of SARS-CoV-2 receptor ACE2 – implications for microvascular inflammation and hypercoagulopathy in COVID-19. BioRxiv. 2020 May.2005.2011.088500. doi: 10.1101/2020.05.11.088500

[6] Varga Z, Flammer AJ, Steiger P, et al. Endothelial cell infection and endotheliitis in COVID-19. Lancet. 2020 May 2;395(10234):1417–1418. doi: 10.1016/S0140-6736(20)30937-5 32325026 PMC7172722

[7] McConnell MJ, Kawaguchi N, Kondo R, et al. Liver injury in COVID-19 and IL-6 trans-signaling-induced endotheliopathy. J Hepatol. 2021 Sep;75(3):647–658. doi: 10.1016/j.jhep.2021.04.050 33991637 PMC8285256

[8] Kang S, Tanaka T, Inoue H, et al. IL-6 trans-signaling induces plasminogen activator inhibitor-1 from vascular endothelial cells in cytokine release syndrome. Proc Natl Acad Sci U S A. 2020 Sep 8;117(36):22351–22356. doi: 10.1073/pnas.2010229117 32826331 PMC7486751

[9] Li X, Sun X, Carmeliet P. Hallmarks of Endothelial Cell Metabolism in Health and Disease. Cell Metab. 2019 Sep 3;30(3):414–433. doi: 10.1016/j.cmet.2019.08.011 31484054

[10] Paneni F, Beckman JA, Creager MA, Cosentino F. Diabetes and vascular disease: pathophysiology, clinical consequences, and medical therapy: part I. Eur Heart J. 2013 Aug;34(31):2436–43. doi: 10.1093/eurheartj/eht149 23641007 PMC3743069

[11] Vanhoutte PM, Shimokawa H, Feletou M, Tang EH. Endothelial dysfunction and vascular disease - a 30th anniversary update. Acta Physiol (Oxf). 2017 Jan;219(1):22–96. doi: 10.1111/apha.12646 26706498

[12] Huang PL. eNOS, metabolic syndrome and cardiovascular disease. Trends Endocrinol Metab. 2009 Aug;20(6):295–302. doi: 10.1016/j.tem.2009.03.005 19647446 PMC2731551

[13] Atochin DN, Huang PL. Role of endothelial nitric oxide in cerebrovascular regulation. Curr Pharm Biotechnol. 2011 Sep;12(9):1334–42. doi: 10.2174/138920111798280974 21235451 PMC3196792

[14] Blum A, Giladi M, Weinberg M, et al. High anti-cytomegalovirus (CMV) IgG antibody titer is associated with coronary artery disease and may predict post-coronary balloon angioplasty restenosis. Am J Cardiol. 1998 Apr 1;81(7):866–8. doi: 10.1016/s0002-9149(98)00019-8 9555776

[15] Du Y, Zhang G, Liu Z. Human cytomegalovirus infection and coronary heart disease: a systematic review. Virol J. 2018 Feb 6;15(1):31. doi: 10.1186/s12985-018-0937-3 29409508 PMC5801777

[16] Badawi A, Di Giuseppe G, Gupta A, Poirier A, Arora P. Bayesian network modelling study to identify factors influencing the risk of cardiovascular disease in Canadian adults with hepatitis C virus infection. BMJ Open. 2020 May 5;10(5):e035867. doi: 10.1136/bmjopen-2019-035867 PMC7228556 32371519

[17] Hemmat N, Ebadi A, Badalzadeh R, Memar MY, Baghi HB. Viral infection and atherosclerosis. Eur J Clin Microbiol Infect Dis. 2018 Dec;37(12):2225–2233. doi: 10.1007/s10096-018-3370-z 30187247

[18] Gattone M, Iacoviello L, Colombo M, et al. Chlamydia pneumoniae and cytomegalovirus seropositivity, inflammatory markers, and the risk of myocardial infarction at a young age. Am Heart J. 2001 Oct;142(4):633–40. doi: 10.1067/mhj.2001.118118 11579353

[19] Nieto FJ, Adam E, Sorlie P, et al. Cohort study of cytomegalovirus infection as a risk factor for carotid intimal-medial thickening, a measure of subclinical atherosclerosis. Circulation. 1996 Sep 1;94(5):922–7. doi: 10.1161/01.cir.94.5.922 8790026

[20] Warren-Gash C, Hayward AC, Hemingway H, et al. Influenza infection and risk of acute myocardial infarction in England and Wales: a CALIBER self-controlled case series study. J Infect Dis. 2012 Dec 1;206(11):1652–9. doi: 10.1093/infdis/jis597 23048170 PMC3488196

[21] Peretz A, Azrad M, Blum A. Influenza virus and atherosclerosis. QJM. 2019 Oct 1;112(10):749–755. doi: 10.1093/qjmed/hcy305 30605546

[22] Ackermann M, Verleden SE, Kuehnel M, et al. Pulmonary Vascular Endothelialitis, Thrombosis, and Angiogenesis in Covid-19. N Engl J Med. 2020 Jul 9;383(2):120–128. doi: 10.1056/NEJMoa2015432 32437596 PMC7412750

[23] Lee J, Sayed N, Hunter A, et al. Activation of innate immunity is required for efficient nuclear reprogramming. Cell. 2012 Oct 26;151(3):547–58. doi: 10.1016/j.cell.2012.09.034 23101625 PMC3506423

[24] Sayed N, Wong WT, Ospino F, et al. Transdifferentiation of human fibroblasts to endothelial cells: role of innate immunity. Circulation. 2015 Jan 20;131(3):300–9. doi: 10.1161/CIRCULATIONAHA.113.007394 25359165 PMC4309381

[25] Meng S, Zhou G, Gu Q, Chanda PK, Ospino F, Cooke JP. Transdifferentiation Requires iNOS Activation: Role of RING1A S-Nitrosylation. Circ Res. 2016 Oct 14;119(9):e129–e138. doi: 10.1161/CIRCRESAHA.116.308263 27623813 PMC5065398

[26] Lai L, Reineke E, Hamilton DJ, Cooke JP. Glycolytic Switch Is Required for Transdifferentiation to Endothelial Lineage. Circulation. 2019 Jan 2;139(1):119–133. doi: 10.1161/CIRCULATIONAHA.118.035741 30586707 PMC6311718

[27] Dal-Pra S, Hodgkinson CP, Dzau VJ. Induced cardiomyocyte maturation: Cardiac transcription factors are necessary but not sufficient. PLoS One. 2019 Oct 17;14(10):e0223842. doi: 10.1371/journal.pone.0223842 31622977 PMC6797484

[28] Inciardi RM, Lupi L, Zaccone G, et al. Cardiac Involvement in a Patient With Coronavirus Disease 2019 (COVID-19). JAMA Cardiol. 2020 Jul 1;5(7):819–824. doi: 10.1001/jamacardio.2020.1096 32219357 PMC7364333

[29] Guo T, Fan Y, Chen M, et al. Cardiovascular Implications of Fatal Outcomes of Patients With Coronavirus Disease 2019 (COVID-19). JAMA Cardiol. 2020 Jul 1;5(7):811–818. doi: 10.1001/jamacardio.2020.1017 32219356 PMC7101506

[30] Shi S, Qin M, Shen B, et al. Association of Cardiac Injury With Mortality in Hospitalized Patients With COVID-19 in Wuhan, China. JAMA Cardiol. 2020 Jul 1;5(7):802–810. doi: 10.1001/jamacardio.2020.0950 32211816 PMC7097841

[31] Stephan BCM, Harrison SL, Keage HAD, Babateen A, Robinson L, Siervo M. Cardiovascular Disease, the Nitric Oxide Pathway and Risk of Cognitive Impairment and Dementia. Curr Cardiol Rep. 2017 Aug 11;19(9):87. doi: 10.1007/s11886-017-0898-y 28801790 PMC5554286

[32] Stone J, Hangge P, Albadawi H, et al. Deep vein thrombosis: pathogenesis, diagnosis, and medical management. Cardiovasc Diagn Ther. 2017 Dec;7(Suppl 3):S276–S284. doi: 10.21037/cdt.2017.09.01 29399531 PMC5778510

[33] National Center for Biotechnology Information [Internet]. Bethesda, MD: US National Library of Medicine; c2021. The Surgeon General’s Call to Action to Prevent Deep Vein Thrombosis and Pulmonary Embolism; 2008 [cited 2021 Oct 25]. Available from: https://www.ncbi.nlm.nih.gov/books/NBK44178/

[34] Ren B, Yan F, Deng Z, et al. Extremely High Incidence of Lower Extremity Deep Venous Thrombosis in 48 Patients With Severe COVID-19 in Wuhan. Circulation. 2020 Jul 14;142(2):181–183. doi: 10.1161/CIRCULATIONAHA.120.047407 32412320

[35] Wichmann D, Sperhake JP, Lütgehetmann M, et al. Autopsy Findings and Venous Thromboembolism in Patients With COVID-19: A Prospective Cohort Study. Ann Intern Med. 2020 Aug 18;173(4):268–277. doi: 10.7326/M20-2003 32374815 PMC7240772

[36] Escher R, Breakey N, Lämmle B. Severe COVID-19 infection associated with endothelial activation. Thromb Res. 2020 Jun;190:62. doi: 10.1016/j.thromres.2020.04.014 32305740 PMC7156948

[37] Monteil V, Kwon H, Prado P, et al. Inhibition of SARS-CoV-2 Infections in Engineered Human Tissues Using Clinical-Grade Soluble Human ACE2. Cell. 2020 May 14;181(4):905–913.e7. doi: 10.1016/j.cell.2020.04.004 32333836 PMC7181998

[38] Teuwen LA, Geldhof V, Pasut A, Carmeliet P. COVID-19: the vasculature unleashed. Nat Rev Immunol. 2020 Jul;20(7):389–391. doi: 10.1038/s41577-020-0343-0 32439870 PMC7240244

[39] Mortus JR, Manek SE, Brubaker LS, et al. Thromboelastographic Results and Hypercoagulability Syndrome in Patients With Coronavirus Disease 2019 Who Are Critically Ill. JAMA Netw Open. 2020 Jun 1;3(6):e2011192. doi: 10.1001/jamanetworkopen.2020.11192 32501489 PMC7275245

[40] Hanff TC, Mohareb AM, Giri J, Cohen JB, Chirinos JA. Thrombosis in COVID-19. Am J Hematol. 2020 Dec;95(12):1578–1589. doi: 10.1002/ajh.25982 32857878 PMC7674272

[41] Lowe GDO. Virchow’s triad revisited: abnormal flow. Pathophysiol Haemost Thromb. 2003 Sep-2004 Dec;33(5–6):455–7. doi: 10.1159/000083845 15692260

[42] National Center for Biotechnology Information [Internet]. Bethesda, MD: US National Library of Medicine; c2021. Rumbaut RE, Thiagarajan P. Arterial, Venous, and Microvascular Hemostasis/Thrombosis; 2008 [cited 2021 Oct 25]. Available from: https://www.ncbi.nlm.nih.gov/books/NBK53453/

[43] Rajeeva NK, Walther BK, Suresh R, Cooke JP, Jain A. Microengineered Human Vein-Chip Recreates Venous Valve Architecture and Its Contribution to Thrombosis. Small. 2020;16(49):2003401. doi: 10.1002/smll.202003401 PMC7791597 33205630

[44] Pandian, NKR, Mannino RG, Lam WA, Jain A. Thrombosis-on-a-chip: Prospective impact of microphysiological models of vascular thrombosis. Curr Opin Biomed Eng. 2018 Mar;5:29–34. doi: 10.1016/j.cobme.2017.12.001 34765849 PMC8580137

[45] Diaz JA, Obi AT, Myers DD, et al. Critical review of mouse models of venous thrombosis. Arterioscler Thromb Vasc Biol. 2012 Mar;32(3):556–62. doi: 10.1161/ATVBAHA.111.244608 22345593 PMC3292052

[46] Bazigou E, Makinen T. Flow control in our vessels: vascular valves make sure there is no way back. Cell Mol Life Sci. 2013 Mar;70(6):1055–66. doi: 10.1007/s00018-012-1110-6 22922986 PMC3578722

[47] Diaz JA, Saha P, Cooley B, et al. Choosing a Mouse Model of Venous Thrombosis. Arterioscler Thromb Vasc Biol. 2019 Mar;39(3):311–318. doi: 10.1161/ATVBAHA.118.311818 30786739

[48] Ingber DE. Reverse Engineering Human Pathophysiology with Organs-on-Chips. Cell. 2016 Mar 10;164(6):1105–1109. doi: 10.1016/j.cell.2016.02.049 26967278

[49] Benam KH, Dauth S, Hassell B, et al. Engineered in vitro disease models. Annu Rev Pathol. 2015;10:195–262. doi: 10.1146/annurev-pathol-012414-040418 25621660

[50] Jain A, Graveline A, Waterhouse A, Vernet A, Flaumenhaft R, Ingber DE. A shear gradient-activated microfluidic device for automated monitoring of whole blood haemostasis and platelet function. Nat Commun. 2016 Jan 6;7:10176. doi: 10.1038/ncomms10176 26733371 PMC4729824

[51] De Ceunynck K, Peters CG, Jain A, et al. PAR1 agonists stimulate APC-like endothelial cytoprotection and confer resistance to thromboinflammatory injury. Proc Natl Acad Sci U S A. 2018 Jan 30;115(5):E982–E991. doi: 10.1073/pnas.1718600115 29343648 PMC5798377

[52] Jain A, Barrile R, van der Meer AD, et al. Primary Human Lung Alveolus-on-a-chip Model of Intravascular Thrombosis for Assessment of Therapeutics. Clin Pharmacol Ther. 2018 Feb;103(2):332–340. doi: 10.1002/cpt.742 28516446 PMC5693794

[53] Mathur T, Singh KA, Pandian NKR, et al. Organ-on-chips made of blood: endothelial progenitor cells from blood reconstitute vascular thromboinflammation in vessel-chips. Lab Chip. 2019 Jul 23;19(15):2500–2511. doi: 10.1039/c9lc00469f 31246211 PMC6650325

[54] Luna DJ, Pandian NKR, Mathur T, et al. Tortuosity-powered microfluidic device for assessment of thrombosis and antithrombotic therapy in whole blood. Sci Rep 2020 Apr 1;10(1):5742. doi: 10.1038/s41598-020-62768-4 32238835 PMC7113244

[55] Rubin R. As Their Numbers Grow, COVID-19 “Long Haulers” Stump Experts. JAMA. 2020 Oct 13;324(14):1381–1383. doi: 10.1001/jama.2020.17709 32965460

[56] Marshall M. The lasting misery of coronavirus long-haulers. Nature. 2020 Sep;585(7825):339–341. doi: 10.1038/d41586-020-02598-6 32929257

[57] WHO.int [Internet]. Geneva, Switzerland: World Health Organization; c2021. A clinical case definition of post COVID-19 condition by a Delphi consensus; 2021 Oct 6 [cited 2021 Nov 10]. Available from: http://apps.who.int/iris/bitstream/handle/10665/345824/WHO-2019-nCoV-Post-COVID-19-condition-Clinical-case-definition-2021.1-eng.pdf.

[58] Mehta JL, Bavineni M. Premature birth, infections, and atherosclerotic cardiovascular disease. Eur Heart J. 2019 Oct 14;40(39):3275. doi: 10.1093/eurheartj/ehz441 31324911

[59] Bavineni M, Wassenaar TM, Agnihotri K, Ussery DW, Lüscher TF, Mehta JL. Mechanisms linking preterm birth to onset of cardiovascular disease later in adulthood. Eur Heart J. 2019 Apr 7;40(14):1107–1112. doi: 10.1093/eurheartj/ehz025 30753448 PMC6451766

[60] Miller JE, Hammond GC, Strunk T, et al. Association of gestational age and growth measures at birth with infection-related admissions to hospital throughout childhood: a population-based, data-linkage study from Western Australia. Lancet Infect Dis. 2016 Aug;16(8):952–61. doi: 10.1016/S1473-3099(16)00150-X 27052469

[61] Bekkering S, Miller JE, Burgner DP. Childhood infection may mediate the relationship between suboptimal intrauterine growth, preterm birth, and adult cardiovascular disease. Eur Heart J. 2019 Oct 14;40(39):3273–3274. doi: 10.1093/eurheartj/ehz438 31324913

[62] West HW, Juonala M, Gall SL, et al. Exposure to parental smoking in childhood is associated with increased risk of carotid atherosclerotic plaque in adulthood: the Cardiovascular Risk in Young Finns Study. Circulation. 2015 Apr 7;131(14):1239–46. doi: 10.1161/CIRCULATIONAHA.114.013485 25802269

